# Sensory Overresponsivity, Loneliness, and Anxiety in Taiwanese Adults with Autism Spectrum Disorder

**DOI:** 10.1155/2018/9165978

**Published:** 2018-09-02

**Authors:** Ya-Cing Syu, Ling-Yi Lin

**Affiliations:** ^1^Department of Occupational Therapy, College of Medicine, National Cheng Kung University, Tainan, Taiwan; ^2^Institute of Allied Health Sciences, College of Medicine, National Cheng Kung University, Tainan, Taiwan

## Abstract

**Purpose:**

Sensory overresponsiveness is highly prevalent in individuals with autism spectrum disorders (ASD) and typically persist into adulthood. However, the role of sensory processing difficulties in influencing emotional well-being among adults with ASD remains unclear. Additionally, the associations between sensory overresponsiveness, anxiety, and loneliness are yet to be examined among adults with ASD. Therefore, to address these critical gaps in the literature, we aimed to investigate the relationships among sensory avoiding, anxiety, and loneliness in a sample of adults with ASD.

**Participants:**

Seventy adults (age, 20–39 years) completed three self-reported measures. All participants had a documented diagnosis of ASD and had normal IQ.

**Methods:**

Sensory processing, loneliness, and anxiety were assessed with the Adult Sensory Profile, short-form UCLA loneliness scale, and Beck Anxiety Inventory, respectively.

**Results:**

Autism spectrum traits and sensory avoiding were associated with anxiety and loneliness. Participants who experienced sensory avoiding more frequently reported higher anxiety and feelings of loneliness, with loneliness mediating the relationship between sensory avoiding and anxiety. More anxiety symptoms in participants with greater sensory avoiding were associated with their higher level of loneliness.

**Conclusion:**

This study demonstrates that the relationships existed between sensory processing difficulties, loneliness, and anxiety among adults with ASD. Interventions regarding sensory processing difficulties and emotional well-being are substantial need for adults with ASD, and our results highlight the importance of sensory overresponsiveness and anxiety in evaluating and improving the psychological well-being of adults with ASD.

## 1. Introduction

Autism spectrum disorder (ASD) is a developmental disorder characterized by problems with communication and social interaction and repetitive body movements or behaviors [1]. Atypical sensory processing is considered a core feature of ASD, and sensory features (hyperactivity or hypoactivity or unusual interest to sensory input) have been included in the criteria for ASD in the fifth edition of the *Diagnostic and Statistical Manual of Mental Disorders* [1].

People experience and process the sensory stimuli from the environment and the body itself continually. Dunn constructed a model using the relationship between neuroscience and behavior concepts to illustrate the way that people manage, process the stimuli, and elicit responses [2]. Every individual has a unique sensory processing pattern [3]. Individual responses to sensory inputs present as a bell-shaped distribution, with most of the people exhibiting moderate responses to sensory stimuli and few people responding more or less intensively [3]. Not all the stimuli can produce responses; the stimuli have to exceed the neurological threshold to activate the nerve cells or the nervous system [3]. With low neurological thresholds, people frequently sense most of the stimuli and trigger the response; on the contrary, people with high neurological thresholds will often overlook the stimuli that others may notice [3]. Another critical construct is the behavior pattern that people perform after they take in and process the sensory inputs [2]. A person may use a passive or active self-regulatory strategy reacting to the things happening around them [3]. Using the interaction of neurological threshold continuum and behavior response continuum, Dunn introduced the four quadrants of sensory processing patterns, including low registration, sensory seeking, sensory sensitivity, and sensory avoiding [2].

According to Dunn's model [3], low registration refers to a passive behavioral response pattern (unable to detect the stimuli that most other individuals have noticed) that is related to a high neurological threshold (require more intense sensory stimuli inputs to generate a response). Sensory seeking refers to an active behavioral response pattern (actively searching or seeking for sensory stimuli) that is related to a high neurological threshold. Sensory sensitivity refers to a passive behavioral response pattern that is related to a low neurological threshold (requires lower magnitude sensory stimuli inputs to generate a response). Individuals in this category may exhibit unpleasant feelings or negative reactions to certain sensory stimuli. Sensory avoidance refers to an active behavioral response pattern that is related to a low neurological threshold. Individuals in this category may establish their own routines or ritual behaviors to reduce their exposure to stimuli [3].

Additionally, Miller et al. [4] indicated that sensory sensitivity and sensory avoiding are two types of sensory overresponsivity patterns. Brown and Dunn [5] and Pfeiffer et al. [6] hypothesized that individuals who overrespond to sensory stimuli have low neurological thresholds, such that minimal stimuli provoke a strong reaction. Thus, sensory overresponsivity refers to an exaggerated or negative response to sensory stimuli. Individuals may experience unpleasant feelings or pain and may perform avoidance or hypervigilance behaviors [4].

Unusual sensory processing negatively influences the daily lives of people with ASD. For example, sensory overresponsivity was highly correlated with sleep problems in children and adolescents with ASD [7, 8]. Another study that looked at sensory processing patterns in higher education students with ASD reported that auditory sensitivity might affect the student's ability to absorb information in a study environment [9]. Students also described sensory preferences related to their choice of leisure activity and social life, and these were linked to lower levels of social skills and adaptive behavior [9]. Pfeiffer et al. [6] found that hypersensitivity was inversely associated with community use and social skills in children and adolescents with Asperger's disorder.

Sensory overresponsivity is common in children and adolescents with ASD. It has been linked to higher levels of anxiety [6, 10, 11] and together with anxiety predicts an increasing number of gastrointestinal problems [12]. Although ASD is a lifelong condition persisting into adulthood, most studies have focused on the pediatric population. Only few studies that included adults with ASD have shown that sensory overresponsivity persists into adulthood [9, 13]. Sensory overresponsivity is prevalent in adulthood and is present in each sensory modality (vision, hearing, touch, smell, taste, and proprioception) among adults with ASD [13]. Moreover, previous findings suggest that overresponsiveness to sensory stimuli is correlated with psychological well-being in children [6, 7, 12, 14]. However, little is known about this issue in adults with ASD, and, therefore, it warrants further investigation.

Anxiety is one of the most common psychiatric symptoms in adults with ASD. According to a recent report, autistic adults are more likely to have an anxiety disorder and behavioral challenges [15]. There is growing interest in the relationship between sensory overresponsivity and anxiety in this population. It has been observed that sensory overresponsivity emerges before anxiety and positively predicts the subsequent increasing levels of anxiety in toddlers with ASD [14]. Similarly, another study found a strong, positive association between anxiety and sensory overresponsivity in children with ASD, but not adults [12]. Although the underlying mechanism that connects sensory overresponsivity to anxiety is not understood, individuals with low neurological thresholds may be uncomfortable with sensory stimuli from the surroundings, which might induce feelings of unease, worry, or anxiety.

Another problematic condition associated with anxiety in ASD is loneliness. Loneliness is a subjective and negative experience and may affect an individual's self-esteem, social functioning, and mental and physical health. Individuals with ASD may face social isolation because of their difficulties with social skills, leading to deeper feelings of loneliness. Many studies have explored the connection between loneliness and external factors, such as friendship and social support; however, research on the association of loneliness and mental health problems in adults with ASD is limited. Evidence suggests that loneliness correlates with increased depression and anxiety in adults with ASD, even after controlling for the effect of ASD symptoms. This suggests that loneliness may be a consequence of social deficits or negative emotional well-being [16].

Considering the high proportion of sensory overresponsivity and anxiety in individuals with ASD, it is logical to speculate that the relationship between sensory overresponsivity and anxiety also manifests in adults with ASD. Adults with ASD who have sensory avoiding or sensory sensitivity may feel overwhelmed by the sensory stimuli, resulting in aggressive or negative reactions. These extreme sensory-responsive patterns may negatively affect the experience of interacting with others or the environment, further elevating feelings of loneliness [17]. Loneliness as a potential mediator might be related to the higher level of anxiety [18, 19]. Compared to Western countries, individuals in Eastern countries are more likely to feel lonely because they pay more attention to interpersonal bonds and their existing social network [20]. Thus, sensory processing and loneliness may result in negative emotional well-being in this population, but critical gaps exist in the literature about this issue, which the current study seeks to address. The present study is aimed at exploring relationships between sensory processing, loneliness, and anxiety in a sample of adults with ASD.

## 2. Materials and Methods

### 2.1. Procedures

The principal investigator has obtained the Institutional Review Board of National Cheng Kung University Hospital approval for the research (A-BR-101-074). We recruited adults with ASD from the local autism groups and organizations. The principal investigator explained all study procedures to the participants. Written informed consent was gathered from all participants.

### 2.2. Participants

The sample size for a linear multiple regression was estimated using G^∗^Power (version 3.1.2) [21]. With a medium effect size (*f*^2^ = 0.15), an alpha of 0.05, a test power of 0.80, and two predictors, the minimum sample size would be 68 participants to achieve adequate power. The statistical power was 0.81.

The characteristics of study participants are presented in Table 1. Participants were adults with ASD (*n* = 70, 46 men and 24 women; mean age: 27.8 years; range: 20–39 years). All participants had a documented diagnosis of ASD by a registered psychiatrist. Individuals had been diagnosed with pervasive developmental disorders using the DSM-IV-TR criteria [22] of autistic disorder, Asperger's disorder, or pervasive developmental disorder not otherwise specified should be diagnosed with ASD [1]. The principal investigator reviewed the disability certificates issued by Taiwan Department of Social Welfare to confirm diagnosis of ASD. The exclusion criterion was marked intellectual disabilities (IQ < 70). To determine participants' reading and writing skills, we asked them to read a newspaper paragraph and then immediately write five of the words they had read. All participants with ASD could read and write Mandarin Chinese.

### 2.3. Measures

#### 2.3.1. Sensory Profile

The Chinese version of the Adult Sensory Profile, which is reliable and valid, was used to examine patterns of sensory processing [23]. It consists of 60 items, which is divided into four quadrants of sensory processing: sensation seeking (an intensive craving for sensory stimuli), sensory sensitivity (overresponsive reactions to sensory input), sensation avoiding (a tendency to reduce stimulation), and low registration (a tendency to not notice everyday sensory events and to respond slowly to them). The score range for each quadrant is 15–75. Sensory overresponsive individuals are hypersensitive and tend to avoid certain sensations. In contrast, sensory-underresponsive individuals are hyposensitive and often engage in sensation-seeking behaviors. Higher scores indicate that certain sensory patterns occur more frequently than others.

#### 2.3.2. Anxiety

The Beck Anxiety Inventory (BAI) was used for measuring the severity of anxiety [24]. The Chinese version has strong construct validity and an excellent reliability [25]. The BAI consists of 21 items that measure the severity of anxiety symptoms in adults. Participants were asked to answer the question “Did you feel anxious this past week?” using a scale of 0 to 3 (0 = “not at all,” 3 = “severely”). The BAI consists of four factors: (1) neurophysiological; (2) subjective; (3) autonomic; and (4) panic. The scores range from 0 to 63, with higher scores indicating more severity of anxiety symptoms. The scores are categorized as follows: minimal level of anxiety (0–7), mild anxiety (8–15), moderate anxiety (16–25), and severe anxiety (26–63). On the Chinese version of the BAI, a cut-off score of 14 was used to discriminate between individuals with and without anxiety [25].

#### 2.3.3. Loneliness

The short-form UCLA loneliness scale (ULS-8) assessed the extent of loneliness and social isolation. A total of 8 items were selected from the revised UCLA loneliness scale [26]. The ULS-8 demonstrates high internal reliability and good validity [27]. Each item was scored on a 4-point Likert scale ranging from 1 (never) to 4 (always). The scale generates loneliness score in the range of 8–32, with higher scores indicating higher degrees of loneliness and greater social isolation.

#### 2.3.4. Autism Spectrum Quotient

The Taiwanese Chinese translation [28] of the autism spectrum quotient (AQ) [29] was used to quantify the autistic traits. The Chinese version demonstrates adequate test-retest reliability and validity [28]. The AQ contains 50 questions divided into five domains: social skills, communication, imagination, attention to detail, and attention switching. The scores range from 0 to 50, with higher scores indicating greater level of autistic traits.

#### 2.3.5. Demographic Information

Participants were asked to report the demographic information including age, sex, comorbid psychiatric disorders, education, employment status, marital status, smoking habits, and drinking habits.

### 2.4. Data Analysis

SPSS 17.0 for Windows (SPSS Inc., Chicago, IL, USA) was used in the analysis of the demographic data and outcome measures for the study variables. Normality was assessed using the Shapiro-Wilk test of normality. We performed Pearson's correlations and multivariate regression analysis to examine relationships between all variables. Statistical significance was set at *p* < 0.05.

## 3. Results

The mean (standard deviation [SD]) scores for the four sensory quadrants (sensory sensitivity, sensation avoiding, sensation seeking, and low registration) were 43.2 (9.7), 45.9 (8.2), 43.6 (8.1), and 41.6 (8.6), respectively. They scored in more than most people in the quadrants of sensation avoiding (35.7%), sensory sensitivity (27.1%), sensation seeking (22.9%), and low registration (21.4%). The mean (SD) score for anxiety was 21.2 (11.9). Using the cut-off score of 14, we determined that more than 70% of the participants (77.9%) reported moderate or severe level of anxiety. The mean (SD) score for loneliness was 21.9 (4.9) on the ULS-8, with about one-third of the participants (32.9%) having scores over 24. The Shapiro-Wilk normality test suggested that loneliness was normally distributed (*p* = 0.083). The median and the range of the three measures can be found in Table 1.

The correlation matrix for the study variables was used to evaluate relationships between study variables. There were significant correlations among sensory avoiding, anxiety, and loneliness (Table 2). A significant positive correlation was observed between sensory avoiding and anxiety, suggesting that participants reported greater anxiety when sensory avoiding occurred more frequently. Sensory avoiding was positively correlated with loneliness, such that the higher the level of reported sensory avoiding, the greater the severity of loneliness. Moreover, participants reported a greater anxiety severity when they experienced greater loneliness.

Separate multiple regression analysis models were applied to investigate whether the sensory processing and autism spectrum traits were associated with levels of anxiety and loneliness (Table 3). Regression analyses indicated that the autism spectrum traits (*β* = .268, *p* = 0.039) and sensory avoiding (*β* = .362, *p* = 0.035) were significant predictors of anxiety (*R*^2^ = 0.16, *F*(5, 64) = 2.50, *p* = 0.039). Autism spectrum traits (*β* = .345, *p* = 0.004) and sensory avoiding (*β* = .413, *p* = 0.009) were significantly associated with loneliness (*R*^2^ = 0.29, *F*(5, 64) = 5.28, *p* < 0.001).

**Figure 1:**
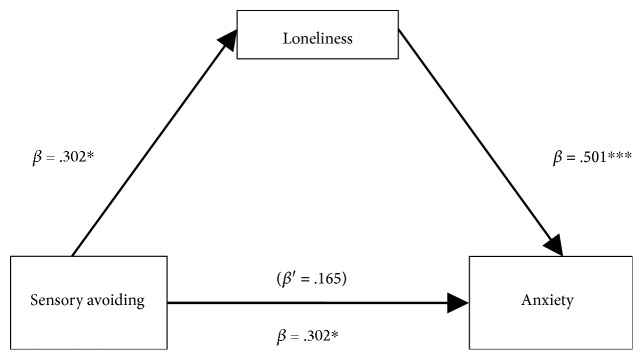
Results of testing for mediation by loneliness on relationship between sensory avoiding and anxiety. Note: ^∗^*p* < 0.05, ^∗∗∗^*p* < 0.001. *β*' referred to standardized coefficient after controlling for anxiety. Type of mediation = full, Sobel's *z* value = 2.178, *p* = 0.0294. Standardized coefficient of sensory avoiding on anxiety: direct = 0.165, indirect = 0.137.

The method outlined by Hayes [30] was followed to examine whether loneliness was a mediating variable that accounts for the relationship between sensory avoiding and anxiety. This hypothesis was tested by a series of regression analysis. Sensory avoiding was significantly associated with loneliness and anxiety (Table 2). After controlling the relation between the loneliness and anxiety, the significant relationship between sensory avoiding and anxiety was reduced or became nonsignificant. A mediation effect would be supported.  Figure 1 shows that loneliness was a mediator that accounts for the relationship between sensory avoiding and anxiety. Analyses using Sobel's test [31] indicated that the mediation effect was supported. This figure indicates that the higher level of anxiety in participants with greater sensory avoiding was mediating by their higher level of loneliness.

## 4. Discussion and Conclusion

The purpose of the present study was to investigate the relationship among sensory avoiding, anxiety, and loneliness in adults with ASD. The results indicate that in adults with ASD, more sensory avoiding was associated with higher reported anxiety and feelings of loneliness. In addition, loneliness served as a mediator between sensory avoiding and anxiety, indicating that adults with ASD reported high levels of anxiety if high sensory avoiding was accompanied by greater loneliness.

Consistent with previous research [13], sensory processing difficulties not only affect children with ASD but also persist into adulthood. In our sample, more than 25% of participants scored more than most people in the sensation avoiding and sensory sensitivity quadrants. This result is partially consistent with that of a study by Clince et al. [9], which revealed that adults with ASD scored higher than most people in the quadrants of sensory avoidance, sensory sensitivity, and low registration. However, contrary to our findings, their participants did not score more than most people in the sensation seeking quadrant. This discrepancy may be attributable to the age difference of the participants; the participants of the previous study were mostly 18–23 years old, whereas the average age of our participants was 28 years. The sensory feature may vary according to age, but this hypothesis warrants further investigation.

Adults with ASD who reported a higher level of sensory avoiding are also experiencing more feeling of loneliness in the present study. Furthermore, loneliness was a significant mediator of the relationship between sensory avoiding and anxiety. To our knowledge, no previous study has demonstrated that loneliness is a mediator of the relationship between sensory processing and anxiety. Our results extend the prior findings in children and adolescents to adults, with a more holistic view of psychological well-being [6, 12, 32]. It is possible that adults with ASD were sensitive to sensory stimuli, which may lead to them avoiding the sensory stimuli encountered in an environmental or social context, resulting in social isolation or loneliness. The negative experience may cause uncomfortable feelings or even anxiety when interacting with the environment or others [33]. Moreover, the lack of strategies for dealing with sensory processing difficulties could also affect their emotional status.

Increased anxiety was significantly associated with loneliness. Cumulative evidence indicates that loneliness has a negative effect on physical health and psychosocial well-being in the general population [34, 35]. The findings from studies of individuals with ASD are highly consistent [16, 32, 36]. Adults with ASD may be more prone to anxiety if they experience more sensory overresponsiveness and perceive greater loneliness. Thus, our results confirm that feelings of loneliness have considerable impacts on the psychosocial well-being of adults with ASD.

The present study does have some limitations. We only included adults with ASD with an IQ above 70, i.e., high-functioning adults with ASD. Due to the inclusion criteria, the percentage of participants with a college education is slightly high. This sample might not be representative of the general adult population with ASD. Additionally, because of the heterogeneity of ASD, the findings of our study may not apply to all adults with ASD. Further research with larger and more diverse sample size from a broader community is required to arrive at conclusions that are more generally applicable. Another limitation is that we only investigated the relationships among anxiety, loneliness, and sensory processing. Recent research reported that adults with ASD were less likely to have friends and more likely to live at home than adults without an ASD diagnosis [15]. Because social networks include family, peers, and friends, future research should consider the influence of external factors on loneliness, such as intimate relationships with others, amount of social support, depression, and living at home vs. living alone. Furthermore, the cross-sectional survey may exhibit bias. Future research should focus on the longitudinal effects of the sensory processing difficulties and emotional well-being of adults with ASD. Despite the limitation, the present findings have important implications for the evaluation of sensory issues and subsequent therapeutic interventions in adults with ASD.

To the best of our knowledge, this study is the first to investigate the relationships among sensory processing, loneliness, and anxiety in Taiwanese adults diagnosed with ASD. It is clinically important to consider the sensory components and emotional status of adults with ASD in their daily lives to effectively evaluate and improve their psychological well-being. Appropriate therapeutic interventions targeting sensory processing issues may have beneficial effects on loneliness and further reduce or prevent anxiety in adults with ASD. Further research should prospectively examine changes in sensory processing and emotional well-being by performing a longitudinal survey and also investigate the relationships among the sensory response and social and emotional functioning in greater detail.

## Figures and Tables

**Table 1:** Demographic characteristics.

Characteristics	Mean ± SD or *n* (%)	Median	Range
Gender			
Male	46 (65.7%)		
Female	24 (34.3%)		
Age (years), mean ± SD	27.8 ± 5.0	27.0	20–39
Autism spectrum quotient	32.9 ± 6.0	34.0	20–43
Social skills	7.0 ± 2.3	7.0	2–10
Attention switching	6.9 ± 1.7	7.0	3–9
Attention to detail	6.3 ± 1.9	6.0	2–10
Communication	6.5 ± 1.8	6.0	1–10
Imagination	6.3 ± 1.6	6.0	1–9
Educational level			
High school and below	16 (22.9%)		
College and above	54 (77.1%)		
Employed	33 (47.1%)		
Marital status: single	70 (100%)		
Comorbid psychiatric disorders	15 (21.4%)		
Sensation sensitivity	43.2 ± 9.7	43	22–67
Sensation avoidance	45.9 ± 8.2	44	33–69
Sensation seeking	43.6 ± 8.1	44	27–64
Low registration	41.6 ± 8.6	43	23–66
Anxiety	21.2 ± 11.9	20	2–43
Loneliness	21.9 ± 4.9	22	11–31

**Table 2:** Intercorrelations of sensory processing, anxiety, and loneliness.

Variables	AQ	Sensation sensitivity	Sensation avoidance	Sensation seeking	Low registration	Anxiety	Loneliness
AQ	−						
Sensation sensitivity	0.221	−					
Sensation avoidance	0.008	0.709^∗∗∗^	−				
Sensation seeking	−0.224	0.244^∗^	0.291^∗^	−			
Low registration	0.173	0.636^∗∗∗^	0.517^∗∗∗^	0.447^∗∗∗^	−		
Anxiety	0.248^∗^	0.217	0.302^∗^	0.061	0.232	−	
Loneliness	0.381^∗^	0.221	0.305^∗^	−0.109	0.274^∗^	0.501^∗∗∗^	−

^∗^ *p* < 0.05, ^∗∗^*p* < 0.01, and ^∗∗∗^*p* < 0.001. AQ = autism spectrum quotient.

**Table 3:** Multiple regressions of autism spectrum traits and sensory processing on anxiety and loneliness (standardized coefficients).

Variables	Anxiety	Loneliness
AQ	0.268^∗^	0.345^∗∗^
Sensation sensitivity	−0.163	−0.262
Sensation avoidance	0.362^∗^	0.413^∗∗^
Sensation seeking	0.012	−0.203
Low registration	0.097	0.258
*R* ^2^	0.16^∗^	0.29^∗∗∗^

^∗^ *p* < 0.05, ^∗∗^*p* < 0.01, and ^∗∗∗^*p* < 0.001.

## Data Availability

The data used to support the findings of this study are available from the corresponding author upon request.
